# Clopidogrel in Critically Ill Patients

**DOI:** 10.1002/cpt.878

**Published:** 2017-11-03

**Authors:** Christian Schoergenhofer, Eva‐Luise Hobl, Peter Schellongowski, Gottfried Heinz, Walter S. Speidl, Jolanta M. Siller‐Matula, Monika Schmid, Raute Sunder‐Plaßmann, Thomas Stimpfl, Matthias Hackl, Bernd Jilma

**Affiliations:** ^1^ Department of Clinical Pharmacology Medical University of Vienna Vienna Austria; ^2^ Department of Medicine I Hematology, and Oncology, Medical University of Vienna Vienna Austria; ^3^ Department of Medicine II Cardiology, Medical University of Vienna Vienna Austria; ^4^ Department of Medicine III Gastroenterology, and Hepatology, Medical University of Vienna Vienna Austria; ^5^ Department of Laboratory Medicine Medical University of Vienna Vienna Austria; ^6^ TAmiRNA GmbH Vienna Austria

## Abstract

Only limited data are available regarding the treatment of critically ill patients with clopidogrel. This trial investigated the effects and the drug concentrations of the cytochrome P450 (CYP450) activated prodrug clopidogrel (*n* = 43) and the half‐life of the similarly metabolized pantoprazole (*n* = 16) in critically ill patients. ADP‐induced aggregometry in whole blood classified 74% (95% confidence intervals 59–87%) of critically ill patients as poor responders (*n* = 43), and 65% (49–79%) responded poorly according to the vasodilator‐stimulated phosphoprotein phosphorylation (VASP‐P) assay. Although the plasma levels of clopidogrel active metabolite normally exceed the inactive prodrug ∼30‐fold, the parent drug levels even exceeded those of the metabolite 2‐fold in critically ill patients. The half‐life of pantoprazole was several‐fold longer in these patients compared with reference populations. The inverse ratio of prodrug/active metabolite indicates insufficient metabolization of clopidogrel, which is independently confirmed by the ∼5‐fold increase in half‐life of pantoprazole. Thus, high‐risk patients may benefit from treatment with alternative platelet inhibitors.

**Study Highlights**

**WHAT IS THE CURRENT KNOWLEDGE ON THE TOPIC?**

☑ Only limited data are available on the effects and on drug concentrations of clopidogrel in critically ill patients. High on treatment platelet reactivity (HTPR) occurs in –30–40% of stable patients.

**WHAT QUESTION DID THIS STUDY ADDRESS?**

☑ We hypothesized that HTPR is frequent in critically ill patients and measured drug concentrations of clopidogrel, its active metabolite, and the similarly metabolized pantoprazole to identify potential mechanisms.

**WHAT THIS STUDY ADDS TO OUR KNOWLEDGE**

☑ Approximately 70% of critically ill patients were insufficiently treated with clopidogrel. The cytochrome‐dependent activation of clopidogrel to its active metabolite is substantially reduced during critical illness. Likewise, the half‐life of pantoprazole, which is also metabolized via cytochrome enzymes, is increased approximately 5‐fold compared with healthy volunteers, indicating a substantially reduced activity of cytochrome enzymes.

**HOW THIS MIGHT CHANGE CLINICAL PHARMACOLOGY OR TRANSLATIONAL SCIENCE**

☑ The results of our trial indicate substantially altered PKs of cytochrome‐dependent drugs in critically ill patients. To optimize treatment, trials in critically ill patients should be included in the drug approval process.

Clopidogrel is a cytochrome (CYP)2C19‐dependent prodrug, which after activation irreversibly inhibits the P2Y12 ADP receptor on platelets.^1^ Some genetic variants of the CYP2C19 enzyme were associated with a reduced activation of the prodrug and consequently with a diminished platelet‐inhibitory response, although conflicting data of the impact on clinical outcomes were reported.^1^, ^2^, ^3^, ^4^ However, besides genetic variants, inflammatory states, and other factors, such as therapeutic hypothermia, may also alter the metabolic activity of CYP enzymes.^5^, ^6^, ^7^ Pro‐inflammatory cytokines reduce the expression and the activity of CYP enzymes and cause a “phenoconversion” of CYP enzymes, a discrepancy between the genetically determined and the actual metabolizing status.^5^ Interestingly, certain microRNAs (miRNAs; i.e., miRNA‐130b) are upregulated during inflammatory responses and decrease expression and activity of CYP enzymes,^8^ whereas low miRNA‐223 levels may be associated with a higher degree of platelet aggregation.^9^

The nonresponsiveness to pharmacological platelet inhibition is termed “high on treatment platelet reactivity” (HTPR),^10^ which is associated with genetic factors and comorbidities, such as diabetes or chronic kidney disease, body weight, and is also caused by drug‐drug interactions.^11^, ^12^, ^13^

Current guidelines recommend the use of acid‐suppressive drugs in ventilated patients to prevent stress ulcers and gastric bleeding.^14^ Therefore, pantoprazole, a proton pump inhibitor, which is also metabolized by CYP2C19 enzymes, is frequently used in critically ill patients.^15^ However, similarly to clopidogrel, genetic variants in the CYP2C19 enzymes influence the pharmacokinetics (PKs) of pantoprazole.^16^

In a small study in patients undergoing successful cardiopulmonary resuscitation who received a loading dose of 600 mg clopidogrel because of a percutaneous coronary intervention, bioavailability of clopidogrel and consequently platelet inhibition were reduced compared with stable patients.^17^ However, there is a lack of data on the effects of clopidogrel in critically ill patients. The objective of this study was to investigate the prevalence of HTPR and to determine drug concentrations in patients admitted to an intensive care unit (ICU). We hypothesized that HTPR may occur frequently due to impaired activation of clopidogrel as a consequence of decreased CYP metabolism by inflammation. To estimate the contribution of altered metabolism, we used an intravenous bolus of pantoprazole as a probe drug. Finally, we analyzed the plasma levels of two miRNAs, miRNA‐130b and miRNA‐223, in patients to investigate a potential association between post‐transcriptional regulation of CYP to CYP metabolism and platelet aggregation.

## RESULTS

Forty‐three clopidogrel‐treated patients and 16 pantoprazole‐treated patients admitted to three medical ICUs participated in this study between November 15, 2012, and September 29, 2016 (**Supplementary Figure S1**). Five patients participated in both groups. **Table** 1 presents the demographics and baseline data of all patients. At 24 h, only 37 patients remained available in the clopidogrel group, because 3 patients were discharged from the ICU and 3 patients died during the short course of the study. Patients in the clopidogrel group were included in the trial 6 days (3–10, median and quartiles) after admission to the ICU. In the pantoprazole group, one patient died during the study day and the 24 h blood sample was not available. The ICU mortality was 44% for clopidogrel‐treated patients and 40% for pantoprazole‐treated patients.

**Table 1:** Demographics and patient characteristics

Parameter	Clopidogrel *n* = 43	Pantoprazole *n* = 16
Gender, m:f	31:12	10:6
Age, years	69 (63–75)	65 (47–73.5)
Body mass index, kg/m^2^	27.8 (24.7–30.2)	24.6 (23.1–27.8)
Hemoglobin, g/dL	9.3 (8.8–10.4)	9.2 (8.7–9.8)
Platelets, G/L	175 (141–258)	184 (88–538)
Leucocytes, G/L	10.7 (8.6–14.3)	12.7 (10.2–14.5)
C‐reactive protein, mg/dL	12.8 (7.7–22.8)	12.1 (9.1–17.7)
SAPS III	62 (52–69)	65 (49–80)
SOFA score	8 (5–11)	10 (5–13)
Mechanical ventilation, no. (%)	31 (72)	14 (88)
ICU mortality, no. (%)	17 (40)	7 (44)
Diagnosis		
Cardiopulmonary resuscitation	19	7
Cardiac	12	1
Respiratory	8	6
Other (Stroke, sepsis, rhabdomyolysis, vascular, epilepsy)	4	2
Genotype		
Poor metabolizer	1	1
Intermediate metabolizer	5	0
Extensive metabolizer	20	6
Rapid metabolizer	13	9
Ultrarapid metabolizer	4	0

Medians and (quartiles) are presented.

ICU, intensive care unit; SAPS, Simplified Acute Physiology Score; SOFA, Sequential Organ Failure Assessment.

### Platelet function

Whole blood aggregometry classified 32 of 43 clopidogrel‐treated patients (74%; 95% confidence intervals (CI) 59–87%) as having HTPR according to the predefined cutoff of 46 U. ADP‐induced aggregation was significantly higher compared with patients with stable coronary artery disease (*P* < 0.001).^18^ The results of ADP‐induced platelet aggregation at baseline correlated reasonably well with platelet counts (r = 0.50; *P* = 0.001). Four patients had platelet counts <80*10^9^/L and/or required therapy with extracorporeal membrane oxygenation and, after exclusion of these, the HTPR rate increased to 82% (*n* = 32/39; 95% CI = 67–93%).

**Figure 1:**
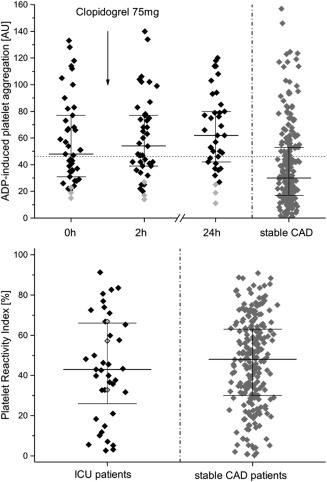
ADP‐induced whole blood aggregometry. Upper panel: ADP‐induced whole blood aggregometry before (0 h) intake of daily 75 mg clopidogrel, as well as 2 h and 24 h thereafter, as well as results from patients with stable coronary artery disease (CAD).^18^ Lower panel: Platelet reactivity index as results of vasodilator‐stimulated phosphoprotein phosphorylation assay in patients in the intensive care unit (ICU) and in patients with stable CAD.^18^ Presented are medians (solid line), quartiles (dashed line), and individual geometric means. The gray symbols show ICU‐patients with platelet counts <75 G/L. The horizontal line shows the cutoff of 46 U and 42%, respectively (*n* = 43 at 0 h and 2 h, *n* = 37 at 24 h).

The vasodilator‐stimulated phosphoprotein phosphorylation (VASP‐P) assay showed that 65% (95% CI = 49–79%) of patients had HTPR with a Platelet Reactivity Index (PRI) > 42% (**Figure** 1).^18^ Compared to stable coronary artery disease no significant difference in the PRI was found.^18^

According to VASP‐P and aggregometry, all poor (*n* = 1) and all intermediate (*n* = 5) metabolizers were found to have HTPR. However, also extensive, rapid, and ultrarapid metabolizers were regularly tested with HTPR (**Supplementary Table S1**). The PRI correlated well with serum cholinesterase levels (r = 0.47; *P* < 0.003).

Whole blood aggregometry and VASP‐P assay supported the diagnosis of HTPR in 70% of patients.

### Concentrations of clopidogrel and its active metabolite

Clopidogrel is rapidly absorbed and metabolized to its active metabolite, which has a short half‐life of ∼0.5–1.0 h. Even after a loading dose of 600 mg, neither clopidogrel nor its active metabolite was detectable in plasma of healthy volunteers after 10 h.^12^

**Figure 2:**
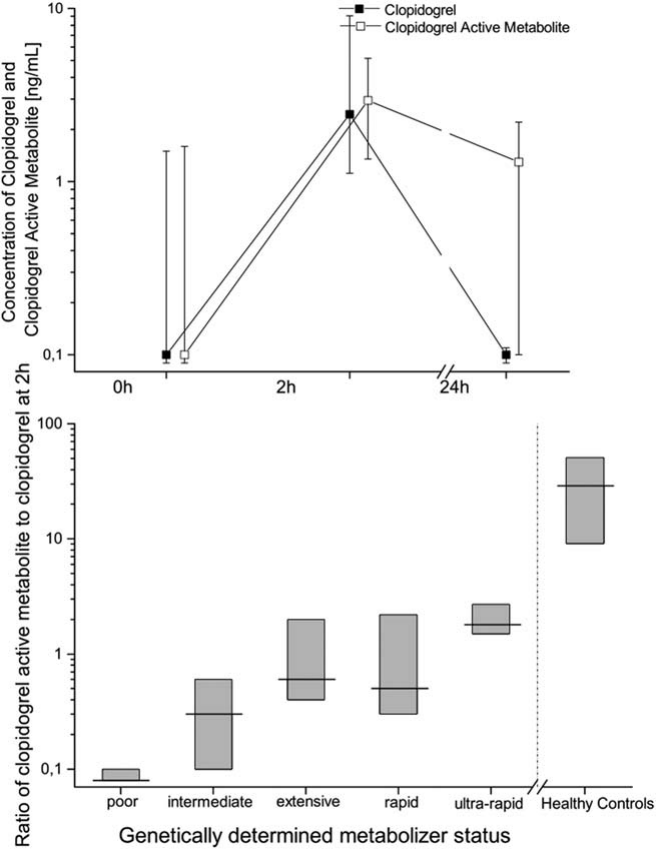
Plasma concentrations of clopidogrel and clopidogrel active metabolite. Presented are medians ± quartiles. Upper panel shows clopidogrel and clopidogrel active metabolite (*n* = 43) before (0 h) and 2 h and 24 h (*n* = 37) after intake of 75 mg clopidogrel. The lower panel shows the ratio of clopidogrel active metabolite concentrations and clopidogrel 2 h after intake of 75 mg clopidogrel subdivided by the different genotypes (*n* = 43) and in comparison with healthy volunteers who received 600 mg clopidogrel.^12^

**Figure** 2 shows the plasma concentrations of clopidogrel and its active metabolite. In six patients, a more detailed analysis was undertaken. This showed a striking increase in the half‐life. In clopidogrel, it increased 10‐fold and in clopidogrel active metabolite it increased 20‐fold (**Table** 2). Trough clopidogrel and active metabolite concentrations were measureable in 30% and 47% of all patients, respectively.

**Table 2:** Pharmacokinetics of clopidogrel and clopidogrel active metabolite (*n* = 6)

	*C_max_*, ng/mL	T_max_, min	T_1/2_, min	AUC_0–∞_ ng*h/mL
Clopidogrel (*n* = 6)				
	29.5 (19.4–56.6)	60 (30–90)	603 (494–1021)	193.6 (10.9–327.7)
Clopidogrel active metabolite (*n* = 6)				
	9.3 (7.8–34)	37.5 (15–90)	12,433 (3,372–13,155)	119.3 (109.1–166.5)

Presented are medians (quartiles).

AUC_0–∞_, area under the curve zero to infinity; *C_max_*, peak plasma concentration; T_1/2_, terminal elimination half‐life; T_max_, time of maximum plasma concentration.

The ratio of clopidogrel active metabolite to clopidogrel concentrations was median 0.6 (quartiles: 0.3–2.0) 2 h after intake of clopidogrel (*n* = 43; **Figure** 2). In healthy volunteers, these ratios were median 28.9 (9.1–50.8) and, therefore, 48‐fold higher (*P* < 0.001).

Neither concentrations of clopidogrel, its active metabolite, nor the ratio of the concentrations correlated with disease scores, laboratory markers of inflammation, ICU mortality, mechanical ventilation, extracorporeal therapy, genotype, or the need for catecholamine therapy.

CYP enzymes are responsible for the activation of the prodrug clopidogrel. Therefore, we investigated the PKs of pantoprazole after intravenous infusion to probe CYP2C19 activity. The plasma concentrations of pantoprazole are presented in **Supplementary Figure S2**. The PKs are listed in **Table** 3. The half‐life increased roughly 5‐fold compared to the expected normal ∼1 h plasma half‐life of pantoprazole.^16^ As a result, trough levels of pantoprazole were measurable in ∼80% of patients.

**Table 3:** Pharmacokinetics of pantoprazole according to genotypes of CYP2C19

		Genotype		
Parameters	All patients (*n* = 16)	Extensive metabolizer (1.1) (*n* = 6)	Poor metabolizer (2.2) (*n* = 1)	Rapid metabolizer (1.17) (*n* = 9)
*C_max_*, ng/mL	2,448 (2,090–2,857)	2,280 (1,617–2,820)	2,845	2,388 (2,111–3,299)
T_max_, min	15 (15–15)	15 (15–15)	15	15 (15–15)
T_1/2_, min	286 (205–433)	433 (320–560)	445	229 (172–307)
AUC_0–∞_, ng*h/mL	7,575 (7,073–11,298)	9,456 (7,145–20,333)	7,302	7,776 (6,770–8,523)

Presented are medians (quartiles).

AUC_0–∞_, area under the curve zero to infinity; *C_max_*, peak plasma concentration; T_1/2_, terminal elimination half‐life; T_max_, time of maximum plasma concentration.

### miRNA concentrations

Plasma concentrations of miRNA‐130b or miRNA‐223 did not correlate with plasma concentrations of clopidogrel, its active metabolite, or PK parameters of pantoprazole. Given the sparse blood sampling and the high variability of PKs in critically ill patients, we also dichotomized patients by measurable trough level concentrations of clopidogrel active metabolite. Intriguingly, miRNA‐130b concentrations were significantly lower in patients with no measureable trough levels when compared with patients with quantifiable plasma concentrations 24 h after the last clopidogrel intake (median = 5.0 (quartiles = 2.3–7.9) vs. 8.9 (4.0–17.6) copies/µL; *P* = 0.015; **Supplementary Figure S3**). Similarly, patients with measurable pantoprazole trough concentrations had significantly higher miRNA‐130b concentrations compared with patients in whom pantoprazole was not detectable 24 h after the last dose (8.2 (7.9–9.5) vs. 18.8 (10.4–33.1) copies/µL; *P* = 0.01; **Supplementary Figure S3**). The miRNA concentrations did not correlate with platelet function assays or markers of inflammation. Levels of miRNA‐130b and miRNA‐223 correlated well with each other (*P* < 0.001; r = 0.66).

## DISCUSSION

This trial in critically ill patients provides several clinically important findings. The majority of patients in the ICU who had previously been receiving a regular 75 mg dose of clopidogrel had high “on treatment” platelet reactivity. To clarify the mechanism, we investigated drug concentrations of clopidogrel. Generation of clopidogrel active metabolite was markedly reduced when compared with data from healthy volunteers or other patient groups.^19^, ^20^, ^21^ Poor metabolism was independently demonstrated by the 4‐fold to sevenfold longer pantoprazole half‐life in critically ill patients, even in rapid and extensive metabolizers, compared with healthy volunteers,^16^ indicating downregulation of CYP2C19 during critical illness.

Although rates of HTPR vary between studies and patient populations, overall, ∼30–40% of patients respond poorly to clopidogrel treatment.^10^, ^19^ Data on clopidogrel concentrations or the effects of clopidogrel in a mixed medical ICU patient population have been lacking. In our trial, the number of poorly responding patients ranged from 65–80%. Therefore, patients treated with clopidogrel who are admitted to the ICU may be at increased risk of having cardiovascular complications. Interestingly, post procedural C‐reactive protein levels were associated with a diminished platelet inhibition to clopidogrel treatment.^22^ A recent study comparing clopidogrel and prasugrel in patients with acute myocardial infarction and cardiogenic shock reported an all‐cause mortality of 51% for clopidogrel‐treated patients and 30% for prasugrel‐treated patients.^23^ Moreover, 17 of 20 clopidogrel‐treated, unstable patients had HTPR according to the VASP‐P assay^24^ after acute myocardial infarction. A retrospective analysis performed in a single‐center stent registry found an overall rate of stent thrombosis of 1.9%, whereas in patients with cardiogenic shock, the risk for definite stent thrombosis increased to 10% with an additional 10% with a probable stent thrombosis.^25^ Our data from critically ill patients demonstrate extraordinary high rates of HTPR in hemodynamically unstable patients and suggest that the underlying pathophysiological problem may be the profoundly reduced concentrations of the clopidogrel active metabolite.

Noteworthy, ADP‐induced whole blood aggregometry and VASP‐P assay supported the diagnosis of HTPR in 70% of patients. In critically ill patients treated with prasugrel, an even greater discrepancy between the two testing systems was reported (65% in ADP‐induced whole blood aggregometry vs. 26% in VASP‐P assay).^26^ Thus, we assume that during critical illness platelets may differ from stable patients and even if the P2Y12 receptor is adequately inhibited, ADP may still induce platelet aggregation, possibly via the P2Y1 receptor.

The PKs of clopidogrel are well characterized in healthy individuals and in less sick patients. After being absorbed, clopidogrel is activated by CYP enzymes and the levels of clopidogrel active metabolite normally exceed that of clopidogrel up to 50‐fold, depending on the genetically determined metabolizer status.^12^, ^21^, ^27^, ^28^ In striking contrast to this 50:1 ratio, the median ratio of the active metabolite to prodrug was only 0.6 (0.3–2.0) in our ICU population 2 h after clopidogrel intake. Even for ultrarapid metabolizers this ratio was only 1.8 (1.5–2.7), which clearly demonstrates an impaired generation of the active metabolite. The parent clopidogrel concentrations in our sick patients in the ICU were similar to an 8‐fold higher dose in healthy volunteers, and 2.5‐fold higher compared to an identical dose in healthy volunteers measured 2 h after intake.^12^, ^27^ These findings suggest that the activation of the prodrug clopidogrel by CYP enzymes is severely impaired in critically ill patients. Additionally, clopidogrel absorption may be reduced due to compromised gastrointestinal motility and dysfunction, which could be, in part, mediated by opiates.^12^ This may also explain the surprisingly long apparent half‐life (∼200 h) of the active metabolite measured in 6 patients in our study compared to a half‐life of 0.5–1 h in healthy volunteers.^21^

To disentangle overlapping poor absorption and metabolism, we determined the PKs of intravenous pantoprazole, which is also metabolized by CYP2C19. We hypothesized that a reduced activity of CYP enzymes should result in an increased half‐life. The half‐life for extensive metabolizers was ∼7 h and for rapid metabolizers ∼4 hours, which is 5‐fold and 4‐fold longer when compared with healthy volunteers with the same genotype.^16^

It was demonstrated previously that miRNA‐130b may be a negative regulator of CYP enzymes.^8^ Intriguingly, miRNA‐130b concentrations were significantly higher in patients who had measurable trough plasma concentrations of clopidogrel active metabolite or pantoprazole. Given the high variability in the PKs of critically ill patients and the sparse blood sampling for clopidogrel‐treated patients, the analysis of trough plasma levels may be especially suitable for assessing CYP activity. Thus, miRNA‐130b may indeed play a role in the downregulation of CYP activity in critically ill patients. However, the magnitude of this effect still needs to be established.

In contrast with stable patients with acute coronary syndromes,^9^ we did not observe any association of miRNA‐223 with platelet function tests. Furthermore, concentrations of miRNA‐130b and miRNA‐223 did not correlate with biomarkers of inflammation. The complexity of each critically ill patient, including prolonged inflammatory responses and different anti‐inflammatory treatments, infectious and noninfectious complications, comorbidities, and the limited sample size may explain the missing associations in our trial. Within each patient, miRNA‐130b and miRNA‐223 concentrations correlated well with each other, indicating common regulatory pathways.

The population included in this trial were indeed critically ill, as reflected by the high mortality of ≥40%. Although the concept of inflammation‐induced “phenoconversion” of CYP enzymes with a consecutively reduced activity has been examined in other patient populations,^5^ such considerations rarely affect treatment in critically ill patients. Our data clearly indicate that besides genetic factors inflammatory states must also be taken into account to optimize therapy for critically ill patients who currently are at risk of being overdosed or underdosed with CYP‐depending drugs.

There are a number of limitations that need to be considered. First of all, to minimize blood loss in anemic critically ill patients, we only performed sparse blood sampling in 37 of 43 clopidogrel‐treated patients. Due to the noninterventional character of the trial, only reference to external control groups simultaneously treated at our center can be made, which, however, is deemed adequate for PK comparisons and because of the large body of published data on HTPR rates with the applied assay systems in different cardiovascular patients, including our previous trials. The trial included a single poor metabolizer and only five intermediate and four ultrarapid metabolizers. However, this was due to chance as we did not power the study to include a certain number of patients with each genotype. The overall sample size was small. However, the observed differences are large and the 95% CIs for HTPR are narrow. Therefore, we deem the sample size to be sufficient. The miRNA‐223 levels were measured in serum samples, which may have limited its discriminative power. Direct comparisons of miRNA‐223 levels to other trials should not be performed.

In conclusion, the number of patients responding poorly to clopidogrel treatment is extraordinarily high in critically ill patients. The PKs of clopidogrel and pantoprazole demonstrate that a reduced activity of CYP2C19 enzyme is at least in part responsible, and whose activity may be downregulated by miRNAs. The critically ill may benefit from treatment with alternative P2Y12 inhibitors.

## METHODS

The Independent Ethics Committee of the Medical University of Vienna and the competent authorities approved the study, which complied with the principles set forth in the Good Clinical Practice guideline and the Declaration of Helsinki. The trial was registered in publicly available databases (EudraCT‐nr. 2012‐002226‐76 and www.clinicaltrials.gov NCT02285751).

All conscious patients gave their informed consent before inclusion in the study. However, because this study included critically ill patients, not all patients were able to give their informed consent before participation in the study. In these patients, the Ethics Committee waived consent.

All patients were admitted to one of three ICUs of the General Hospital of Vienna.

### Patients

The patients in the ICU were >18 years of age, admitted to a medical ICU with prior clopidogrel (75 mg Plavix tablets; Sanofi, France) or pantoprazole (40 mg intravenous bolus; Takeda, Germany) treatment, were included in the trial. Exclusion criteria included allergies or hypersensitivities to the trial drugs, active bleeding, known coagulation disorders, or intake of other antiplatelet drugs except for acetylsalicylic acid. Included patients were on long‐term clopidogrel treatment for coronary artery disease or received daily doses of 40 mg intravenous pantoprazole as a prophylaxis for stress ulcers.

### Study design

Blood samples were drawn before patients received the daily dose of 75 mg clopidogrel (administered either orally or via a nasogastric tube). Samples were then drawn again 2 and 24 h postdrug administration. A more detailed PK analysis was obtained from 6 subjects taking 75 mg clopidogrel and in 16 subjects receiving 40 mg pantoprazole intravenously. In those patients, blood samples were obtained at the following time points: 0, 15 min, 30 min, 45 min, 1 h, 2 h, 4 h, 6 h, and 24 h. Clopidogrel tablets had to be crushed and/or dissolved in 0.9% sodium chloride solution, if they were administered via a nasogastric tube. Pantoprazole was infused as an intravenous bolus. Blood samples were drawn using existing central venous or arterial lines. Plasma samples were harvested after centrifugation at 2000 g for 10 min at 4 °C, and aliquots (500 µL) stored at ‐80 °C.

### Platelet function testing

The primary end point of the trial was ADP‐induced whole blood aggregometry. Whole blood aggregation was determined using the Multiple Electrode Aggregometry on the Multiplate Analyzer (Dynabyte Medical). ADP‐induced platelet aggregation was performed, as explained previously (in detail in the **Supplementary Material**).^12^ To define HTPR, we chose the recommended cutoff of >46 U (arbitrary units) for our study.^29^

The VASP‐P assay was measured by an enzyme linked immune assay, as described previously (see also **Supplementary Material**).^30^ Platelet reactivity is expressed as the PRI. A PRI cutoff of >42% predicted ischemic events most sensitively and was, therefore, chosen as a cutoff for HTPR in this study.^31^

The results of platelet reactivity tests of patients with stable coronary artery disease treated with daily clopidogrel, which have been published elsewhere,^18^ were included as a control group.

### Pharmacokinetics, genetic polymorphisms, and miRNA quantification

Plasma concentrations of clopidogrel and pantoprazole were assessed using liquid chromatography tandem mass spectrometry.^12^, ^32^ The PKs were calculated using the commercially available software Kinetica 2000 (version 3.0; InnaPhase, Philadelphia, PA). All subjects were genotyped for CYP2C19 polymorphisms, as described previously.^33^ Patients with a 2/2 polymorphism were classified as poor metabolizers, those with a 1/2 polymorphism as intermediate metabolizers, those with a 1/1 polymorphism as extensive metabolizers, those with a 1/17 polymorphism as rapid metabolizers, and those with a 17/17 polymorphism as ultrarapid metabolizers.

The ratios of the active metabolite to the prodrug were calculated. As a control group, we included these ratios measured in an earlier trial in healthy volunteers who received a 600 mg clopidogrel loading dose, which have been published elsewhere.^12^

The concentrations of miRNA‐130b and miRNA‐223 were quantified in serum by quantitative polymerase chain reaction using components of the ThrombomiR kit (TAmiRNA, Austria) that is based on a previously published method^34^ (in detail see **Supplementary Material** online and **Supplementary Figure S4**). These miRNAs were chosen based on their association with cytochrome enzymes and platelet aggregation.^8^, ^9^

### Disease scores and clinical data

The Sequential Organ Failure Assessment (SOFA) score and the Simplified Acute Physiology Score (SAPS) III were calculated using the official SAPS 3 Score Calculation Sheets from www.saps3.org on trial day 1. Clinical data was obtained from chart review.

### Statistical analysis

Initially, we planned to screen up to 100 patients to identify 36 patients with HTPR for inclusion in a subsequent randomized trial. However, we terminated recruitment early on in that trial because the prevalence of HTPR was very high and physicians were reluctant to randomize patients.

The sample size calculation for pantoprazole was based on previous publications demonstrating a half‐life of 1.27 ± 0.41 h in extensive metabolizers. A sample size of six per subgroup (either extensive or rapid metabolizers) should suffice to show a twofold prolongation in half‐life (i.e., > 3 SDs, with 90% power, and an alpha error of 1%).

Baseline characteristics, demographics, results of platelet function tests, and PKs are presented by descriptive statistics. Nonparametric Spearman correlations were calculated. Commercially available statistical software was used (IBM, SPSS 22, and Microsoft Excel 2010). The influence of genotypes on PKs was compared using nonparametric Spearman correlation.

## CONFLICT OF INTEREST

M.H. is employed at TAmiRNA. J.S.M. received lecture fees from Astra Zeneca, Daiichi Sankyo, Bayer, Eli Lilly, and Roche.

## SOURCE OF FUNDING

This work was supported by the Austrian Science Funds (grant number SFB54P04).

## AUTHORS' CONTRIBUTIONS

C.S. wrote the manuscript. B.J. and C.S. designed the research. B.J., C.S., E.H., P.S., G.H., W.S., J.S.M., M.S., R.S.‐P., T.S., and M.H. performed the research. B.J. and C.S. analyzed the data.

## Supporting information

Supporting Information
