# Supplementary material for: Clopidogrel in Critically Ill Patients
Source: Clin Pharmacol Ther. 2017 Nov 3;103(2):217–23. doi: 10.1002/cpt.878 (PMC5813104; doi:10.1002/cpt.878)
Supplement: Supplementary file 1 — Supporting Information [file CPT-103-217-s001.docx]

**Supplement**:

**Whole blood aggregometry**

Whole blood aggregation was determined using the Multiple Electrode Aggregometry (MEA) on the Multiplate Analyzer (Dynabyte Medical). The system detects the electrical impedance change due to the adhesion and aggregation of platelets on two independent electrode-set surfaces in the test cuvette (1). A 1:2 dilution of whole blood anti-coagulated with heparin and 0.9% NaCl was stirred at 37°C for 3 min in the test cuvettes, ADP (adenosine diphosphate, 6.4μM), or arachidonic acid (AA =0,5 mM) were added and the increase in electrical impedance was recorded continuously for 6 min (1). The mean values of the two independent determinations are expressed in units (U: tenth of area under the curve). The reference values for the test are as follows: ADP 29–118 U (2) and AA 75–130 U (according to the manufacturer's information). A good reproducibility of MEA has been reported (<6% variability) (3).

**Vasodilator-Stimulated-phosphor-Protein Phosphorylation (VASP-P) assay**

The VASP-P enzyme-linked immune-assay (ELISA) was performed as reported previously (4). After activation with PGE_1_ or PGE_1_+ADP, incubation for 10 minutes (min) and lysis, samples were vortexed and stored at -20° degrees Celsius. After thawing at room temperature (RT) samples were vortex-mixed. For antigen immobilization, 180µl of each sample were transferred to the plate and 180µl dilution buffer were pipetted into blank wells, which were covered and incubated for 30 min at RT. The wells were washed three times with each 300ml washing solution. For immobilization of immuno conjugate, 200 ml of diluted specified mouse monoclonal antihuman VASP-P ser 239 antibody coupled with peroxidase was added immediately. The wells were covered, incubated again for 30 min at RT and the washing step was repeated. Color development was performed by adding 200ml tetra-methyl-benzidine and incubating for 5 min at RT. The reaction was stopped with 100ml H2SO4 and a 2-min-incubation-step. Within 4h after stopping the reaction, the absorbance of the reaction product was measured at 450nm. The PRI was calculated using optical density (OD) in the presence of PGE1 alone or PGE and ADP by means of the formula:

PRI(%)= [(OD450_nmPGE1_-OD_450nm(PGE1+ADP )_/(OD450_nmPGE1_-OD_450nmBlank_)]*100

Calculated values fell sometimes below zero in the ELISA. In this case the values were set to zero for all comparisons.

**Determination of Genotype**

DNA was purified according to standard procedures. The analysis of CYP2C19*2, rs4244285 c.681G>A, p.Pro227=,  CYP2C19*3, rs4986893 c.636G>A, p.Trp212*, and CYP2C19*17, rs12248560 c.-806C>T was performed by Real-Time PCR Real-Time PCR Real-Time PCR Real-Time PCR  and melting-curve analysis using the Light Mix Kit CYP2C19*2 and CYP2C19*3, the LightSNiP CYP2C19*17 rs12248560 assay (TibMolbiol, Berlin, Germany), and the LightCycler FastStart DNA Master Hybridisation Probes kit (Roche Diagnostics, Penzberg, Germany) on a Light Cycler 2.0 (Roche) according to the manufacturer’s suggestions.

**Assessment of pharmacokinetics**

*Clopidogrel and active metabolite*

Blood samples (6 mL) were collected in pre-cooled EDTA tubes (BD Vacutainer, Becton Dickinson, Schwechat, Austria). To stabilize the free thiol group of the active clopidogrel metabolite, 37.5 µL of 500 mM 3-methoxyphenacyl bromide (MPB, Sigma-Aldrich, Vienna, Austria) in acetonitrile (Sigma-Aldrich, Vienna, Austria) was added to each of the samples immediately after collection. The blood samples were gently inverted and centrifuged at 2000 x g (15 minutes, 4°C) to separate the plasma. Aliquots were stored at -70°C until analysis. Plasma concentrations of clopidogrel and its active metabolite were determined by liquid chromatography tandem mass spectrometry (LC-MS/MS) based on a published procedure (5, 6).

Analyses were performed using an AB Sciex QTRAP 5500 (AB Sciex, Framingham, US) system.

0.4 mL of an internal standard working solution with 1 ng/mL D_3_-clopidogrel (@rtMolecule, Poitiers, France), and 10 ng/mL MPB derivative of ^13^C_6_-clopidogrel active metabolite (ALSAChim, Strasbourg, France) in acetonitrile was added to 0.2 mL plasma. The precipitated samples were vortexed and centrifuged at 2400 x g. After a 1+1 dilution of the supernatant with water, 5 μL were injected into the LC-MS/MS using the electrospray ionization in positive mode. Chromatographic separation was achieved on a Kinetex Reversed Phase C18 column (particle size 2.6 µm, 50 mm x 3 mm, Phenomenex, Torrance, US) at a column temperature of 55°C using a mixture of methanol/30 mM ammonium formate solution, (68/32, v/v) at a flow rate of 0.05 mL/min. The transitions m/z 322.0 to m/z 212.0 for clopidogrel, m/z 325.1 to m/z 215.1 for D3-clopidogrel, m/z 504.1 to m/z 354.1 for the MPB derivative of clopidogrel active metabolite, and m/z 510.3 to m/z 354.3 for the MPB derivative of 13C6-clopidogrel active metabolite were monitored in multiple reaction monitoring mode.

A calibration curve with plasma samples was constructed for clopidogrel (Syn Fine Research, Ontario, Canada) (0.5, 1, 5, 10, 15 ng/mL), and MPB derivative of clopidogrel active metabolite (ALSAChim, Strasbourg, France) (5, 10, 50, 100, 150 ng/mL), respectively.

*Pantoprazole*

Plasma concentrations of pantoprazole were determined by liquid chromatography tandem mass spectrometry (LC-MS/MS) based on a published procedure (7).

Analyses were performed using an AB Sciex QTRAP 5500 (AB Sciex, Framingham, US) system. 0.2 mL of an internal standard working solution with 500 ng/mL omeprazole (Sigma-Aldrich, St. Louis, US) in methanol was added to 0.05 mL of lithium heparin plasma. The precipitated samples were vortexed and centrifuged at 2400 x g. 1 μL of the supernatant was injected into the LC-MS/MS using the electrospray ionization in positive mode. Chromatographic separation was achieved on a Kinetex Reversed Phase C18 column (particle size 2.6 µm, 50 mm x 3 mm, Phenomenex, Torrance, US) using a mixture of water and methanol (40/60, v/v, both containing 1 ‰ of 5 ml ammonium acetate) at a flow rate of 0.3 mL/min. The transitions m/z 384.0 to m/z 200.0 for pantoprazole and m/z 346.1 to m/z 198.1 for omeprazole were monitored in multiple reaction-monitoring mode.

A calibration curve (50, 100, 200, 500, 1000, 2000, 5000 ng/mL) was constructed in plasma for pantoprazole (Sigma-Aldrich, St. Louis, US).

**microRNA analysis in serum and plasma**

microRNAs were analyzed using components of the thrombomiR™ kit (TAmiRNA, Austria). Serum or plasma samples were thawed on ice and centrifuged at 12,000g for 5 minutes to remove any cellular debris. 200 µL sample were mixed with 1000µL lysis buffer and 1µL synthetic spike-in provided with the kit. After a 10 minutes incubation at room temperature 200µL chloroform were added and samples were centrifuged at 12,000g for 15 minutes at 4°C. 650µL of the upper aqueous phase was taken and mixed with 7µL glycogen (50mg/mL). Samples were transferred to RNA-binding columns, where RNA was precipitated with 750 µL ethanol followed by washing with RPE and RWT buffer. RNA as eluted in 30 µL nuclease free water and stored at -80°C.

In order to remove traces of heparin, which might be present due to heparin-treatment of patients, 5 µL RNA were mixed with 0.25 RiboLock RNAse Inhibitor (Thermo Fisher Scientific, USA) 1.25µL Heparinase 1mg/mL (Sigma, USA) and 3.5µL heparinase buffer (10mM Tris, 25mM NaCl, 2mM CaCl2 pH=7.5). Samples were incubated for 3 hours at 25°C and stored at -80°C. cDNA was synthesized using the thrombomiR™ kit (TAmiRNA, Austria) using 4µL of the heparinase treated RNA. To control enzyme inhibition an RNA spike-in (cel-miR-39-3p) was added to the cDNA synthesis reaction. For qPCR analysis 4µL 1:40 diluted cDNA was mixed with 5 µL Exilent SYBR Green master mix (Exiqon, Denmark) and 1µL LNA-enhanced miRNA primer (Exiqon, Denmark) for UniSp4, cel-miR-39-3p, miR-130b-3p or miR-223-3p. PCR amplification was performed in a Roche LC480 II instrument (Roche, Germany) with the following settings: 95°C for 10 min, 45 cycles of 95°C for 10 s and 60°C for 60s, followed by melting curve analysis. To calculate the cycle of quantification values (Cq-values) the second derivative method was used. Copies/µL were calculated with following formula copies=2^(37-Cq of the sample) according to the assumption that a Cq-value of 37 is one copy/µL. To ensure equal quality of the obtained data UniSp4 and cel-miR-39-3p was used to monitor RNA Isolation and cDNA synthesis (see Figure S4).

Table S1. Distribution of high “on treatment” platelet reactivity over genotypes and platelet function tests

| Test | Genotype | | | | |
| --- | --- | --- | --- | --- | --- |
|  | Poor Metabolizer | Intermediate Metabolizer | Extensive Metabolizer | Rapid Metabolizer | Ultra-rapid Metabolizer |
| MEA-ADP | 1/1 (100%) | 5/5 (100%) | 16/21 (76%) | 7/12 (58%) | 3/4 (75%) |
| VASP-Assay | 1/1 (100%) | 5/5 (100%) | 7/9 (78%) | 6/7 (86%) | 1/1 (100%) |

Table S1. HTPR distribution over genotypes and platelet function tests.

Figure S1. Flowchart of the trial


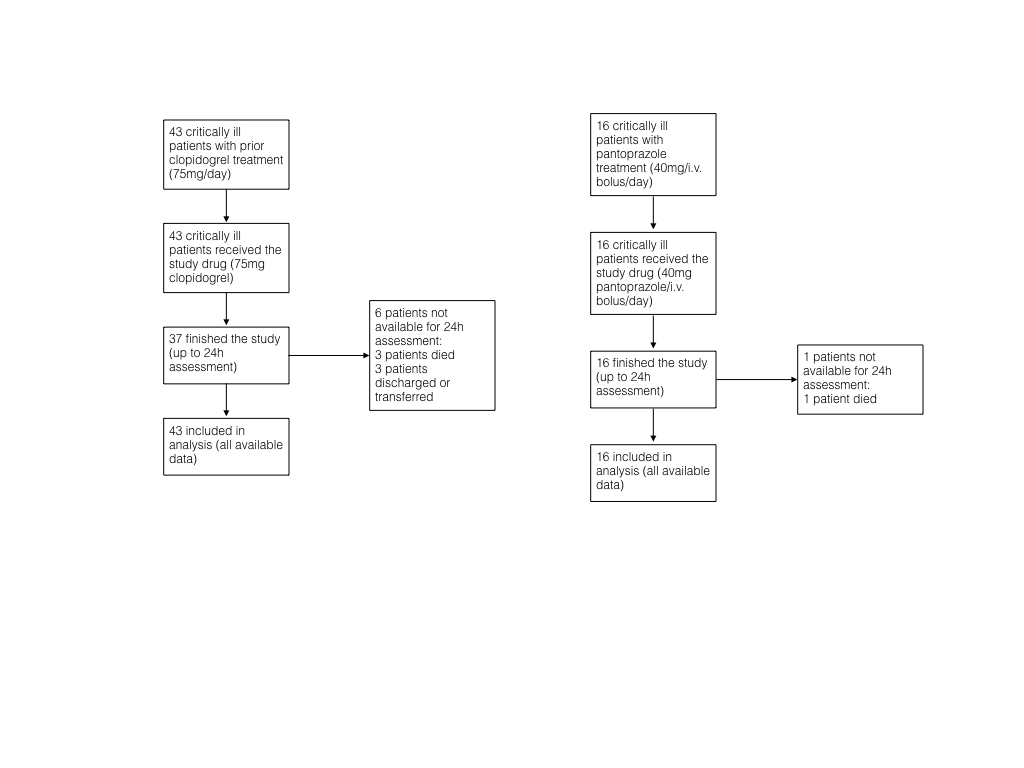


Figure S1 Flowchart of the trial.

Figure S2. Pantoprazole plasma concentrations

Figure S2. Pantoprazole plasma concentrations after bolus infusion of 40mg pantoprazole subdivided by genetically determined metabolizer status (n=16).

Figure S3


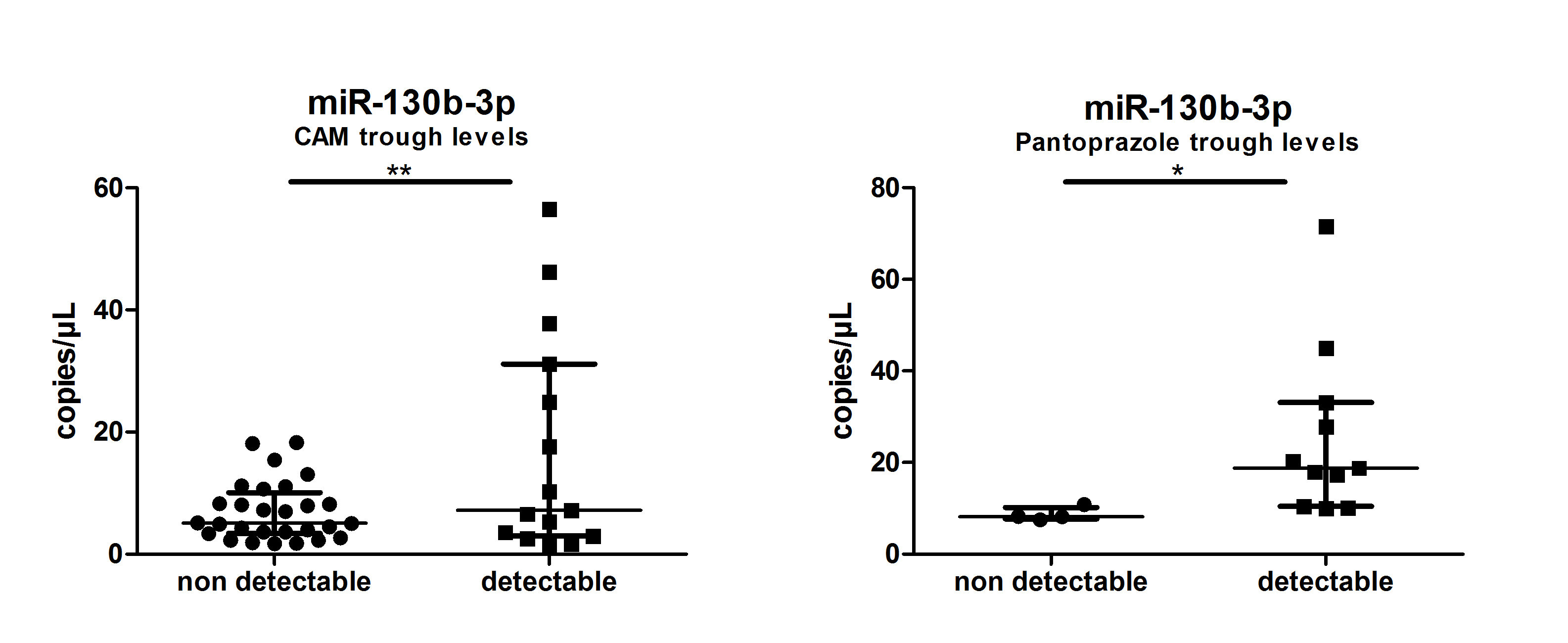


Figure S3. Plasma concentrations of miR-130b

Presented are individual values, means ± standard deviations of miR-130-3p (copies/µL) in patients with measureable trough concentrations of clopidogrel active metabolite (n=43, left panel) or pantoprazole (n=16, right panel).

Figure S4 Spike-in controls

Figure S4. Spike-in quality control data. UniSp4 is added to the workflow prior to RNA extraction to measure the technical variation of the entire workflow. Cel-miR-39-3p (blue) is added to total RNA prior to reverse transcription to measure the variation introduced during synthesis of cDNA.

1. Sibbing D, Braun S, Jawansky S, Vogt W, Mehilli J, Schomig A, et al. Assessment of ADP-induced platelet aggregation with light transmission aggregometry and multiple electrode platelet aggregometry before and after clopidogrel treatment. Thromb Haemost. 2008;99(1):121-6.

2. Mueller T, Dieplinger B, Poelz W, Calatzis A, Haltmayer M. Utility of whole blood impedance aggregometry for the assessment of clopidogrel action using the novel Multiplate analyzer--comparison with two flow cytometric methods. Thromb Res. 2007;121(2):249-58.

3. Toth O, Calatzis A, Penz S, Losonczy H, Siess W. Multiple electrode aggregometry: a new device to measure platelet aggregation in whole blood. Thromb Haemost. 2006;96(6):781-8.

4. Hobl EL, Jilma B, Derhaschnig U, Schoergenhofer C, Schwameis M, Jilma-Stohlawetz P. Comparison of a new ELISA-based with the flow cytometric assay for vasodilator-associated stimulated phosphoprotein phosphorylation to assess P2Y12 -inhibition after ticagrelor intake. Cytometry B Clin Cytom. 2015;88(6):385-8.

5. Takahashi M, Pang H, Kawabata K, Farid NA, Kurihara A. Quantitative determination of clopidogrel active metabolite in human plasma by LC-MS/MS. J Pharm Biomed Anal. 2008;48(4):1219-24.

6. Hobl EL, Stimpfl T, Ebner J, Schoergenhofer C, Derhaschnig U, Sunder-Plassmann R, et al. Morphine decreases clopidogrel concentrations and effects: a randomized, double-blind, placebo-controlled trial. J Am Coll Cardiol. 2014;63(7):630-5.

7. Li Y, Ding MJ, Ma J, Wang S, Wu XL, Xu HJ, et al. Quantification of pantoprazole in human plasma using LC-MS/MS for pharmacokinetics and bioequivalence study. Eur J Drug Metab Pharmacokinet. 2011;35(3-4):147-55.
